# Psychometric properties of the newly translated creole multidimensional scale of perceived social support (MSPSS) and perceived adequacy of resource scale (PARS) and the relationship between perceived social support and resources in Haitian mothers in the US

**DOI:** 10.1186/s40359-016-0113-8

**Published:** 2016-02-09

**Authors:** Jean Hannan, Marise Alce, Adrian Astros

**Affiliations:** Florida International University, University Park, Nicole Wertheim College of Nursing and Health Sciences, 11200 SW 8th Street, AHC 3, Rm 324A, Miami, FL 33199 USA; Jackson Memorial Hospital, 1601 N.W. 12 Avenue, Miami, FL 33136 USA; Florida International University, University Park, 11200 SW 8th Street, Miami, FL 33199 USA

**Keywords:** Psychometrics, Creole, MSPSS, Social support, PARS, Adequacy of resources

## Abstract

**Background:**

Low income postpartum mothers with little to no social support have increased maternal and infant morbidity and mortality, especially those with limited English proficiency and limited accesses to resources. Haitians, a growing minority in the US are an understudied population excluded from most studies due to the lack of instruments in Creole. The most widely used instruments for measuring social support, the Multidimensional Scale of Perceived Social Support (MSPSS) and Perceived Adequacy of Resource Scale (PARS), are not available in Creole. Currently, there are no published studies on the psychometric properties of the MSPSS or the PARS in Creole. Data from Haitian mothers are needed to identify potential postpartum mothers and infants most at risk of developing adverse maternal and infant outcomes from a lack of social support and perceived resources. The purpose of this study is to test the psychometrics of the newly-translated Creole instruments of the MSPSS and PARS with a sample of bilingual (Creole/English) mothers.

**Methods:**

The MSPSS and PARS were translated and back translated from English to Creole. The adapted Creole versions of the instruments were tested using a convenience sample. A total of 85 Haitian mothers’ completed both instruments in Creole and English 2 weeks apart.

**Result:**

Internal consistency reliability and stability were strong for both the MSPSS and PARS (.91–.99). The two instruments had strong reliability and validity for the translated Creole versions and similar to the English versions.

**Conclusion:**

The MSPSS and PARS are a valid measure of perceived social support and resources. Psychometric findings suggest that the newly translated Creole versions are good representations of the English versions indicating the translation process was successful. The newly translated instruments available in Creole provide non-English speaking Haitian mothers the opportunity to participate in studies.

## Background

Postpartum mothers with little to no social support and inadequate resources experience poor physical and emotional maternal and infant health outcomes. This is especially true for those of low incomes, minority status, limited English proficiency and inadequate access to health care [1–6]. Limited social support and resources contribute to postpartum depression, posttraumatic stress disorder, and poor maternal and infant outcomes; this occurs more so in minorities than non-minorities [7–11]. The most common limited social support and resources include: lack of help or support from family, friends, partner or spouse; loss of employment; lack of money; limited access to services; transportation; and isolated conditions amongst others [7, 12, 13]. However, the majority of studies reporting on postpartum women and their perceived social support and resources are focused on mainly White non-minority women, Hispanic women and African American women with little to no studies with Haitian women despite the changing demographics of the US population.

Haitian immigrants and Haitian Americans, tripling in numbers between 1990 and 2012 represent approximately 1.5 % of the total U.S. foreign-born population [14] and are considered one of the fastest-growing minority groups in the US [15]. Despite their growing numbers, Haitians are under-represented in research especially low income postpartum mothers. Data from Haitian mothers are needed to identify potential postpartum mothers and infants most at risk of developing adverse maternal and infant outcomes from a lack of social support and resources. However, research to examine social support and resources in this group is extremely limited due to the lack of instruments in the Creole language. The most widely-used instruments for measuring social support and resources in English are the Multidimensional Scale of Perceived Social Support (MSPSS) and the Perceived Adequacy of Resource Scale (PARS). Currently, there are no published studies on the psychometric properties of the MSPSS or the PARS in Creole. English versions of these instruments have reported strong reliability and validity. The PARS has not been translated into other languages and is only available in English. However, the MSPSS has been translated in many languages including: Spanish, French, Italian; Simplified Chinese, Traditional Chinese and others with reported good reliability and validly [16–19]. Mosqueda et al. [20] examined the psychometrics of the MSPSS Spanish version. Internal consistency and validity was supported by a strong Cronbach’s Alpha (.88) for the total scale and had a positive correlation between the Spanish MSPSS and the Spanish Self-efficacy General support Scale (*r* = .36; *p* ≤ 0,01). By having reliable and valid Creole versions of these instruments would allow this important group of postpartum Haitians mothers to be included in studies examining their social support and adequacy of resources.

### Conceptual basis and development of the instruments

The MSPSS developed by Zimet et al. (1988a) [21] is a 12-item easy to use, self-report instrument used to measure the perceptions and adequacy of social support from 3 sources: family, friends and significant others. In the early development, the MSPSS was constructed with 24-items addressing relationships with family, friends, and significant other. Each item is rated on a 5-point Likert-type scale ranging from *strongly disagree* (1) to *strongly agree* (5). From the results of several pilot studies and repeated factor analyses, items that did not directly address perceived social support and did not form consistent, conceptually clear factors were excluded. The revised and current version of the MSPSS includes 12 items and in attempt to increase response variability and minimize a ceiling effect, a 7-point rating scale ranging from *very strongly disagree* (1) to *very strongly agree* (7) was implemented. Dahlem et al. (1991) [22] tested the revised MSPSS and found it a psychometrically sound instrument. The 12 items on the MSPSS divide into factor groups relating to sources of the support (i.e., Family, Friends, or Significant Other) with four items in each group. Higher summative scores indicate greater perceived social support. The possible scores for the total scale range from 7–84. Total scores ranging from 69–84 indicate high perceived social support; total scores ranging from 49–68 indicate a moderate perceived social support and a total scores ranging from 12–48 indicates a low perceived social support. Research indicates that mothers with low perceived social support scores are at risk for adverse health outcomes such as depression [23, 24]. The MSPSS takes 5–10 min to complete.

During the initial testing, Zimet et al. (1988) [21] administered the MSPSS to a sample of 275 male and female undergraduate students. Validity of the 12-item MSPSS was obtained by factor analysis using the Kaiser Normalization test with oblique rotation demonstrating items had high loadings. In a later study, Zimet et al. (1990) [25] demonstrated factorial validity using Varimax rotation with 3 different types of samples: a sample of 265 pregnant women in their third trimester, a sample of 74 adolescents attending a high school in Europe and a sample of 55 US pediatric residents. All items loaded strongly on the 3 subscales: Family, Friend and Significant Other. Zimet et al. [21] confirmed the validity of the three-subscale structure of the MSPSS in their 1988 study. Construct validity was examined between the MSPSS subscales with depression and anxiety sub scales of the Hopkins Symptom Checklist (HSCL) [26]. Perceived social support from the Family subscale was inversely correlated with depression (*r* = −.24, *p* < .01) and anxiety (*r* = −.18, *p* < .01). Perceived social support from the Friends subscale was inversely correlated with depression symptoms only (*r* = −.24, *p* < .01); whereas, perceived support from Significant Other subscale was significantly inversely correlated to clinical depression (*r* = −.13, *p* < .05). Canty-Mitchell and Zimet (2000) [27] found high internal consistencies and factor analysis confirmation of the instrument’s hypothesized structure, supporting the validity and reliability of the MSPSS in a sample of 237 urban adolescents, the majority female (58.5 %) and an African American (68 %) sample. Trujols et al. (2014) [28] in a sample of 173 men and women (65.1 % women) reported a significant negative correlation but small-to-moderate in magnitude found between the Quick Inventory of Depressive Symptomatology-Self-Report _16_ (QIDS-SR _16_) [29] and the MSPSS (*r* = −.38, *p* < 0.001). Stewart et al. (2014) [30] examined the construct validity in a sample of 583 women attending an antenatal clinic in Malawi. The Self Reporting Questionnaire [31] (a depression screening tools) and MSPSS total was negatively correlated (*r* = −.28; *p* < .001) and the subscales; Significant Other (*r* = −.21; *p* < .001), Family (*r* = −.30; *p* < .001) and Friends (*r* = −.19; *p* < .001) were also negatively correlated supporting its validity. Other studies supported the construct validity of the MSPSS by a moderate negative correlation (*r* = −.35; *p* < .002) between MSPSS and depression scores in a diverse group of 154 students at an urban college reporting high life stress but reporting no correlation (*r* = .02; *p* = ns) with low life stress [22]. Kazarian and McCabe (1991) [32] reported moderate correlations between the MSPSS total score and the Beck Depression Inventory [33] (*r* = −.31; *p* < .05) in 165 university students, the Children’s Depression Inventory [34] (*r* = −.58; *p* < .05) and the Piers Harris Self-Concept scale [35] (*r* = .42; *p* < .05) in a sample of adolescent psychiatric patients.

Zimet et al. (1988a) [21] first tested the internal reliability of the MSPSS on 275 subjects obtaining coefficient alphas that ranged from .85 to .91for the total scale and the 3 subscales indicating good internal reliability. Sixty-nine of the 275 subjects were retested 2–3 months after completing the initial questionnaires. Test-retest values ranged from .72 to .85, indicating good stability. Kuo et al. (2004) [36] reported a high internal consistency with the MSPSS (.87) in their study examining depression in postpartum Hispanic women. Similar findings were reported by Ponizovsky and Ritsner [37] in a study examining loneliness measured by the revised UCLA-loneliness scale among immigrants. Internal consistencies for this study were high for the MSPSS (.75–.83). Nakigudde et al. (2009) [38] in a sample of 240 postpartum mothers from Uganda obtained internal reliability of .83 for the total MSPSS. Stewart et al. (2014) [30] in a study with 583 women attending an antenatal clinic in Malawi reported high internal consistency for the subscales and the total scale (.85–.91). Canty-Mitchell & Zimet (2000) [27] also found high internal consistencies supporting the validity and reliability in a sample of mostly African American adolescents (68 %), the majority female (58.5 %) with Cronbach’s alpha of .93 for the total MSPSS and .91 (Family), .89 (Friends), and .91 (Significant Other) for the subscale.

The Perceived Adequacy of Resources Scale (PARS) developed by Rowland et al. (1985) [39] is a 28- item scale and a 21- item scale. Both versions use a Likert scale that ranges from 1 (strongly disagree) to 7 (strongly agree). The 21-item scale was developed for use in studies with limited time (i.e., telephone interviews). The PARS is designed to measure the perceived adequacy of resources that contribute to the quality of life attained by individuals and families. A perception of inadequate resources represents or reflects limited access to resources. The PARS assesses the adequacy of 7 distinct resources categorized as (1) physical environment, (2) health/physical energy, (3) time, (4) financial, (5) interpersonal, (6) knowledge/skills, and (7) community resources. During its development, seven resource categories were used to construct a set of 35 items pertaining to the perceived adequacy of resources. Items were short, limited to one idea, and consisted of terms that were simple and understandable for use with a wide range of reading ability. All items are worded positively. The initial testing of the PARS was with a sample of 89 subjects who were participants in a resource management study [40]. Factor analysis demonstrated 7 items did not have high loadings and were removed resulting in the 28-item scale. The possible range of scores are from 28 to196. Low perceived adequacy of resources scores range from 28 to 83, moderate perceived adequacy of resources scores range from 84 to140 and high perceived adequacy of resources scores range from 140 to196. Lower scores indicate poor quality of life, barriers to resources and a higher risk of poor health outcomes. The PARS takes 10–15 min to complete.

The 28-item PARS was tested on a sample of 520 men and women (164 men, 356 women) with varying backgrounds (ages 19–82). Construct validity was established by factor analysis using the principal axis method and Varimax rotation demonstrating positive loadings for all 28 items. Seven resource categories were extracted: physical environment, health/physical energy, time, financial, interpersonal, knowledge/skills, and community resources. The shortened version of the scale was developed by selecting three items in each of the seven factors that had the highest loadings and the best approximation of the simple structure. This resulted in the removal of 7 items from the 28-item scale and the creation of the 21-item PARS. Factor analysis for the 21-item scale using the Varimax method resulted in items loading stronger in the first factors. However, only six factors were extracted; three items from each of two categories; Interpersonal and community resources, merged to form one factor. The correlation among the 21-items remained the same. The 21-item scale demonstrated equivalence to the longer version, except the measurement of interpersonal resources was not distinguishable from that of community resources. The validity supported by other researchers had a negative correlation (*r* = −.41, *p* < .01) with the Parenting Stress Index [41] in a sample of 113 mostly Native American mothers (69 %) with young children [42]. Easom, and Quinn (2006) [43] reported a strong negative correlation (*r* = −.60, *p* = 0.18) in a sample of 80 elderly caregivers with the Health Promotion Activities of Older Adults Measure [44]. A study with a sample of 500 Turkish University employees found strong correlations (*r* = .70, *p* < .01; *r* = −.80, *p* < .01) with marital status, number of children and age supporting the construct validity [45].

During the development of the PARS, reliability was supported by a high Cronbach’s alpha for both the 28-item (.89) and the 21-item (.86) indicating good internal consistency [39]. Burrell et al. (1992) [42] reported high internal reliability of .87. Others reported similar Cronbach’s alpha (.87) with rural elderly caregivers exploring folk home remedies [43].

Unfortunately, the Multidimensional Scale of Perceived Social Support (MSPSS) and the Perceived Adequacy of Resources Scale (PARS) are not available in Creole. The purpose of this study was to test and compare the reliability and construct validity of the newly-translated Creole versions and the original English versions of the MSPSS and PARS with a sample of bilingual (Creole/English) mothers.

The study questions were:

1. What are the internal consistency and stability (test-retest) reliabilities of the Creole and English versions of the MSPSS and the PARS at two different time points 2-weeks apart? Is the reliability similar for the Creole and English versions of the MSPSS and PARS at each time point and across time?
2. Are the scores obtained on the Creole and English versions similar at each time point?
3. Are the Creole and English MSPSS and PARS scores at each time point related to number of years living in the US, partner status, number of children, income, and education? Are the correlations of the Creole and English-version similar for both time points and across time points?

## Methods

### Sample

The study was approved by the Internal Review Board of Florida International University. A convenience sample of 85 Haitian mothers was recruited among faculty, staff, and students at Florida International University and their friends using personal contacts. *Inclusion criteria:* Haitian mothers, 18 years of age or older (allowing the instruments to be applicable to most age groups of Haitian mothers), bilingual (English/Creole) and able to read both English and Creole. *Exclusion criteria*: Haitian men, Haitian women without children and not bilingual (Creole/English), were unable to read in both Creole and English or any condition that prohibited completion of the study instruments.

### Measures

The MSPSS and the PARS (28-item version) were administered to Haitian mother participants. The Creole versions of the MSPSS and the PARS were followed by the English versions. All mothers completed both instruments a second time 2 weeks later.

### Number of years living in US

Mothers were asked to indicate the total number of years they have lived in the US.

Partner/marital status mothers were asked to indicate their current by selecting one of the following options: married and living together, married and living alone, separated and living with a partner, separated and living alone, divorced and living with a partner, divorced and living alone, widowed and living with a partner, widowed and living alone, single (never married) and living with a partner, and single and living alone. All options that indicated the participant was living with a partner were coded as “partnered,” and the remaining options were coded as “not partnered.”

Number of children was measured by asking mothers to indicate the number of children they have either living with them or living away from them.

Education was measured by having mothers select their highest level of education completed. The 7 range options went from a low 1 (less than high school) to a high of 7 (doctorate degree).

Annual income was measured by having mothers select the income range that best described their total family income. The 6 options ranged from a low of 1 (less than $10,000/year) to a high of 6 (>$50,000 or more/year).

Translation and back-translation were used to develop the Creole version of the MSPSS and the PARS. All of the items on the MSPSS and PARS were translated into Creole by a Haitian Creole speaking Florida International University (FIU) graduate nursing student. The instruments were then translated back into English by a second Haitian Creole speaking FIU graduate nursing student who had not seen the English version. The back-translated English version and the original English version were compared by the study team for equivalence of meaning. When differences between the two English versions occurred, the study team and the two Creole speaking graduate nursing students discussed the meaning of the original English item. After reaching an agreement, the Creole speaking graduate nursing students approved the Creole wording that most closely related to the meaning of the English item.

### Procedure

Haitian mothers 18 years and older in the South Florida area were recruited for the study. Graduate nursing students in FIU’s Nursing program recruited Haitian mothers through friends, family classmates and from local community places they attended (i.e., churches, social functions). The Graduate nursing students explained the study to the potential mothers, screened for inclusion and exclusion criteria, and answered their questions. Mothers meeting the study criteria and who agreed to participate were asked to sign an informed consent form. Following informed consent, mothers completed the MSPSS and the PARS in both English and Creole and the demographic form in English only. Two weeks after completion of the initial instruments, all mothers completed the same two instruments again for test–retest reliability. All mothers were given a $5 gift card each time they completed their questionnaires. The reliability and validity of the Creole and English versions of the MSPSS and PARS were tested and compared at each time point and test-retest was done over a 2 week period.

## Results

### Sample

Eighty five Haitian mothers between the ages of 20 and 72 years were recruited into the study (see Table 1). The majority of the mothers were born in Haiti (91.8 %) with a mean of 20.4 years living in the US. Most of the mothers were employed (77.6 %), were college graduates educated in Haiti (75.3 %), were partnered (62.4 %), and almost half (43.6 %) earned $30,000 or less annually (43.6 %). Creole was their primary language (see Table 2).

**Table 1:** Sample characteristics (*N* = 85)

Characteristic		
Age [M (SD)]		45.8 (11.1)
Birthplace [n (%)]	Haiti	78 (91.8 %)
	USA	7 (8.2 %)
Education [n (%)]	High School Graduate	21 (24.7 %)
	College Graduate	64 (75.3 %)
Partner status [n (%)]	Partnered	53 (62.4 %)
	Not Partnered	32 (37.6 %)
Number of Children [n (%)]	1 – 3	68 (80.0 %)
	4 or more	17 (20.0 %)
Employment [n (%)]	Employed	66 (77.6 %)
	Not Employed	19 (22.4 %)
Annual Income [n (%)]	< $30,000	37 (43.6 %)
	$40,000 - $59,999	9 (10.6 %)
	$60,000 or more	24 (28.3 %)
	No Answer	15 (17.5 %)
Years living in the US	[M (SD)]	20.4 (12.3)
Percentage of life in the US	45.4 %	

Note: *M* (SD) Mean (standard deviation)

**Table 2:** Language used most often

Daily	Creole	25 (29.4 %)
	English	8 (9.4 %)
	Both	52 (61.2 %)
At Work [n (%)]	Creole	16 (19.0 %)
	English	36 (42.4 %)
	Both	32 (38.6 %)
At Home [n (%)]	Creole	72 (84.7 %)
	English	5 (5.9 %)
	Both	8 (9.4 %)

### Reliability testing

Internal consistency reliability of the Creole and English versions of the MSPSS was supported by strong Cronbach’s alphas that were similar for the newly translated Creole version and the original English version for the total scale and the three subscales. Stability reliability was supported by strong test–retest correlations 2 weeks apart for both the Creole and the English versions. The English correlation result was *r* = .82 and the Creole correlation result is *r* = .87. The internal consistency and stability reliabilities of the newly-translated Creole version of the MSPSS are supported by these results (see Table 3).

**Table 3:** Reliability testing of Creole and English versions

Measure	Type of reliability	Language	Value
Multidimensional Scale of Perceived Social Support	Internal Consistency Time 1	English	.94
		Creole	. 96
	Internal Consistency Time 2	English	.99
		Creole	.93
	Stability Test-retest Correlations	English	*r* = .82^a^
		Creole	*r* = .87^a^
Perceived Adequacy of Resources Scale	Internal Consistency Time 1	English	.91
		Creole	.93
	Internal Consistency Time 2	English	.92
		Creole	.93
	Stability Test-retest Correlations	English	*r* = .92^a^
		Creole	*r* = .94^a^

^a^Pearson Product Moment Correlation

Internal consistency reliability of the Creole and English versions of the PARS Scale was supported by strong Cronbach’s alphas that were the same for the newly translated Creole version and the original English version. Stability reliability was supported by strong test–retest correlations after a 2-week interval, *r* = .92 for English versions and *r* = .94 for the Creole versions. Internal consistency and stability reliabilities of the newly-translated Creole version of the PARS are supported by these results (see Table 3).

### Validity testing

Validity was first examined by comparing the total mean scores of the Creole and English versions the MSPSS and PARS at both time points (see Table 4). In addition, construct validity was examined by hypothesis testing using 5 demographic variables (number of years living in the US; partner status; number of children; education; income) with Time 1 Creole and English versions MSPSS and PARS total scores (see Table 5). It was expected that Haitian women’s perception of social support and perceived adequacy of resources would be effected with the number of years living in the US, they were partnered, the number of children they had, their incomes and level of education. A positive correlation would be expected by a greater length of time living in the US, are partnered, have higher incomes, fewer children and the level of education.

**Table 4:** Validity testing of Creole and English versions MSPSS and PARS

	Time point	English *M (SD)*	Creole *M (SD)*	Paired t-value
MSPSS	T1	55.5 (17.2)	56.2 (17.4)	0.70
	T2	60.5 (17.5)	60.7 (17.0)	0.45
MSPSS Subscales				
Friend	T1	17.6 (6.4)	16.7(6.4)	0.90
	T2	18.3 (6.2)	18.2 (5.9)	0.28
Family	T1	19.3(6.2)	19.1 (6.1)	0.23
	T2	20.5(5.9)	20.6(5.6)	0.12
Significant Other	T1	20.5 (7.1)	20.7(7.2)	0.58
	T2	21.1(6.7)	21.5 (5.8)	0.73
PARS	T1	108.9 (22.5)	103.4 (25.3)	0.84
	T2	94.9 (29.4)	94.3 (29.8)	0.83

Note: *M* (SD) Mean (standard deviation)

**Table 5:** Construct validly MSPSS and PARS: time point 1

Variable		
MSPSS	English	Creole
Years Living in US	*r* = .30, *p* = .01*	*r* = .30, *p* = .01*
Partnered	*r* = .31, *p* = .01*	*r* = .32, *p* = .01*
Number of Children	*r* = .05, *p* = .06	*r* = .06, *p* = .55
Education	*r* = .19, *p* = .09	*r* = .21, *p* = .06
Income	*r* = .26, *p* = .03*	*r* = .30, *p* = .01*
PARS		
Years Living in US	*r* = .11, *p* = .61	*r* = .10, *p* = .67
Partnered	*r* = .44, *p* = .02^*^	*r* = .39, *p* = .05^*^
Number of Children	*r* = −.19, *p* = .35	*r* = −.10, *p* = .64
Education	*r* = .17, *p* = .43	*r* = .27, *p* = .18
Income	*r* = .16, *p* = .44	*r* = .21, *p* = .34

*p value is significant

The results of the means on the English and Creole versions total scores of the MSPSS and the PARS were very similar and the results of the paired *t* tests at both time points were not significantly different. Using Pearson-product moment, correlations of the total scores of the English and Creole versions of the MSPSS and the PARS with the number of years living in the US, partnered, number of children, education and income were compared at each time point to examine the construct validity of the newly translated Creole versions. Although 3 of the 5 correlations were significant, the magnitudes of the correlations for the Creole and English versions of the MSPSS and PARS were similar and all were in the expected direction (Table 5). Two of the variables, Number of Children and Education had a negative correlation but did not the expected magnitude. However, as expected lower income Haitian mothers who had lived in the US the least amount of time and were not parented had a significantly negative correlation perceiving themselves to have little social support and inadequate resources.

## Discussion

The results of this study indicate that the psychometric findings for newly translated Creole Multidimensional Scale of Perceived Social Support and the Perceived Adequacy of Resources Scale are good representations of the English versions. Internal consistency reliability and stability over a 2 week period were strong for the English and Creole versions for both instruments. Stability of this study’s reliability for the English versions was similar to that reported in other studies. Nakigudde et al. (2009) [38] reported reliabilities of the MSPSS in a sample of 240 postpartum mothers of .83. Stewart et al. (2014) [30] in a study with 583 antenatal women reported high internal consistency for the subscales and the total scale (Cronbach’s alpha = .85–.91). Kuo et al. (2004) [36] reported high internal consistency (.87) in postpartum Hispanic women. Others also reported strong reliability for the Multidimensional Scale of Perceived Social Support. [21, 27, 37] Rowland et al. (1995) [39] reported a high Cronbach’s alpha for both the 28 item (.89) and the 21 item (.86) PARS. Burrell et al. (1992) [42] also reported high internal reliability of .87 in a sample of 113 mostly Native American mothers. Others reported strong Cronbach’s alpha (.87) with rural elderly caregivers exploring folk home remedies [43].

Results of this study support the validity of the two versions of both Multidimensional Scale of Perceived Social Support and the Perceived Adequacy of Resources Scale. Total scores on the English and Creole versions of the MSPSS and the PARS were not statistically different at both time points. The Creole and English versions at both time points had similar means and standard deviations. Total scores on the Creole and English versions of the MSPSS and PARS were strongly correlated.

Direction and magnitude of the correlations between the Creole versions of the MSPSS and the PARS with the number of years living in the US, partnered, number of children, education and income were similar to the corresponding correlations for the English versions. There was a significant positive correlation with number of years living in the US and women experiencing increased social support and a positive perception of adequate resources. As expected, partnered mothers reported a significant higher amount social support and perceived resources. Another positive correlation included the mothers’ perceived social support and perceived resources increased with increased income and education. However, an expected negative correlation with the number of children the mother reported lowered her perception of social support and resources.

Our sample of Haitian mothers was mostly partnered, foreign educated and working professionals. However, because they did not have had a US education they are more likely to have lower incomes. Additionally, they may have limited English proficiency which may have an effect on their comprehension of the English items of the MSPSS and the PARS. These participants also did not have any formal education in the Creole language. Formal education in Haiti is in French. To date, the written Creole language is yet to be fully developed [46]. Additionally, Creole, their native language and the one they speak most often makes it possible they are limited English proficient, as do 63 % of immigrants in the US [47].

The Creole-speaking population in South Florida is a good representation of the Haitian people from their country. This allowed the study to have native Creole speakers to participate in the item translation, back-translation, and discussion of the final wording for each item. Testing of the Creole version with other Creole-speaking groups, particularly a younger and less educated Haitian sample is recommended.

## Conclusion

The reliability and validity evidence for our newly translated Creole versions of the MSPSS and the PARS are very good and equivalent to the reliability and validity estimates for the English version, suggesting our translation process were successful. The US is continually experiencing changes in demographics, as seen with the rapid increase in the Haitian population. To date, Creole is not a well develop written language and because of this there are few translated instruments that have been psychometrically tested. Having these instruments available in Creole will allow currently excluded non-English speaking Haitian mothers to participate in studies. Including Creole speaking mothers into studies will provide more accurate data on postpartum support and perceived resources which are needed to improve on maternal and infant health outcomes. This study adds to the few psychometrically sound Creole instruments available for research.

## Abbreviations

MSPSS: multidimensional scale of perceived social support
PARS: perceived adequacy of resource scale
